# CircRNA circ_0006156 inhibits the metastasis of prostate cancer by blocking the ubiquitination of S100A9

**DOI:** 10.1038/s41417-022-00492-z

**Published:** 2022-06-27

**Authors:** Yuwei Zhang, Fengping Liu, Yangkun Feng, Xinyu Xu, Yang Wang, Sha Zhu, Jian Dong, Shanchao Zhao, Bin Xu, Ninghan Feng

**Affiliations:** 1 Department of Urology, Affiliated Wuxi No.2 Hospital, Nanjing Medical University, Wuxi, China; 2 Wuxi School of Medicine, Jiangnan University, Wuxi, China; 3 Medical College of Nantong University, Nantong, China; 4 Department of Oncology, Affiliated Wuxi No.2 Hospital, Nanjing Medical University, Wuxi, China; 5 Department of Urology, The Third Affiliated Hospital of Southern Medical University, Guangzhou, China; 6 Department of Urology, Nanfang Hospital, Southern Medical University, Guangzhou, China; 7 Department of Urology, Affiliated Zhongda Hospital of Southeast University, Nanjing, China

**Keywords:** Prostate cancer, Gene regulation

## Abstract

Circular RNAs (circRNAs) have been demonstrated to play vital roles in cancer development and progression. However, studies on the association between circRNAs and prostate cancer (PCa) are still lacking. CircRNA sequencing of two pairs of PCa tissues and adjacent normal tissues was conducted in the present study, and qRT–PCR was performed to verify the results. Functional experiments were performed to investigate cellular functions after specific changes. Mass spectrometry analysis after RNA pull-down experiments and Co-IP assays were further conducted. Downstream target proteins were predicted via online databases and detected in vitro by Western blot analysis and in vivo by immunohistochemistry. Hsa_circ_0006156 (subsequently named circ_0006156) expresses at low levels in both PCa tissues and cells, and it significantly inhibits the migration and invasion of PCa cells. Circ_0006156 binds to and blocks the ubiquitination of S100A9. Moreover, functional assays revealed that circ_0006156 represses the malignant progression of PCa by binding to S100A9. Finally, in vivo experiments showed that circ_0006156 suppresses PCa migration and invasion by increasing S100A9, revealing circ_0006156 as a potential novel effective target for PCa treatment.

## Introduction

Prostate cancer (PCa) is the second most common tumor of males worldwide, and over one million people suffer from PCa every year [1]. The morbidity and mortality of PCa are increasing year by year, and the overall morbidity currently ranks fourth among all tumors after breast, lung and colorectal cancers [1]. As PCa has a high dependence on androgens, androgen deprivation therapy (ADT) has been an efficient treatment that, in most cases, inhibits tumor progression [2]. However, resistance to ADT inevitably occurs in PCa patients, and the disease develops into castration-resistant prostate cancer (CRPC) [3]. After second-line hormonal therapy for metastatic CRPC patients, the median survival time is ≤16 months, and prostate cancer-specific death eventually occurs [4]. Although previous studies have reported several molecular biomarkers of PCa progression, the discovery of new biomarkers is urgent due to the current challenges of clinical treatment.

In recent years, the importance of noncoding RNAs (ncRNAs) in the occurrence and development of PCa has received increasing attention [5]. Circular RNA (circRNA), which uniquely exhibits a covalently closed loop structure, is a new type of ncRNA derived from precursor mRNA and confers higher stability and potential as a biomarker [6, 7]. CircRNAs can be classified into ecircRNAs composed of only exons, ciRNAs containing only introns and EIciRNAs consisting of both, among which ecircRNAs account for over 80% of discovered circRNAs [8, 9]. Previous studies have shown that circRNAs regulate mRNAs through the competitive endogenous RNA (ceRNA) network in the progression of cancers [10, 11], while recent studies have reported that a few circRNAs can bind to RNA-binding proteins (RBPs) to play biological functions in cancers [12–14]. However, studies of circRNAs and RBPs in PCa are scarce.

S100A9, which was first identified in the context of multiple inflammatory reactions, is now considered as a member of the S100 family of calcium binding proteins [15]. The S100 family consists of more than 20 members and each plays unique roles in signal transduction [16]. S100A9 is a calcium binding protein important in the pathogenesis of different cancer types according to previous studies, such as breast cancer, colon adenocarcinoma, hepatocellular carcinoma, etc [17–19].

In the present study, we found that circ_0006156 is downregulated in both PCa tissues and cells and that it has a negative correlation with the migration and invasion of PCa cells. Mechanistically, circ_0006156 binds to S100A9 and stabilizes the protein to inhibit PCa metastasis by blocking the ubiquitination of S100A9.

## Materials and methods

### Ethical statement and tissue collection

This study was approved by the Ethics Committee of Wuxi No. 2. Hospital affiliated with Nanjing Medical University (2021-Y-80). Forty-four PCa specimens and their paired normal tissues were obtained from patients at Wuxi No. 2. Hospital affiliated with Nanjing Medical University. All patients received no endocrine therapy before surgery, and all patients underwent radical prostatectomy. The tissues were immediately preserved in liquid nitrogen.

### Cell culture

A normal prostate cell line (RWPE-1) and human PCa cell lines (LNCaP, PC3, DU145 and 22RV1) were acquired from the Cell Bank of the Chinese Academy of Sciences (Shanghai, China). All cells were cultured in RPMI 1640 medium (Gibco, USA) supplemented with 10% fetal bovine serum (BI, Israel) and 1% penicillin/streptomycin (Gibco, USA) in an atmosphere of 5% CO_2_ at 37 °C. All cells were mycoplasma-free cells.

### Total RNA extraction and quantitative real-time PCR (qRT–PCR)

TRIzol reagent (Invitrogen, USA) was used to extract total cellular RNA, and reverse transcription was then performed with HiScript III RT SuperMix (Vazyme, China) for circRNA and mRNAs. qRT–PCR was then performed using HieffTM qPCR SYBR® Green Master Mix (Yeasen, China) and a LightCycler® 96 SW 1.1 system (Roche, Switzerland). ACTB was used as an endogenous control, and the comparative cycle threshold value (2^-ΔΔCT^) was used to calculate the results. The sequences of primers can be seen in Supplemental Table 2.

### Western blot assay

RIPA lysis buffer (Beyotime, China) containing protease inhibitor (Beyotime) was used to extract total cellular protein. Total proteins were separated by sodium dodecyl sulfate–polyacrylamide gel electrophoresis and then transferred to a polyvinylidene difluoride (PVDF) membrane. The PVDF membrane was then blocked with 10% milk for 2 h followed by incubation with the indicated primary antibody (1:1000, Abcam, USA) overnight at 4 °C. After incubation with the corresponding secondary antibody (1:5000, Abcam) at room temperature for 1 h, the protein bands were visualized using enhanced chemiluminescence (Tanon, China). For all WB images, quantitative density analyses were performed using Image J (version 1.47).

### Oligonucleotide transfection and construction of stably transfected cells

The oligonucleotides to suppress or overexpress circ_0006156 and S100A9 were designed by RiboBio (Guangdong, China). The lentivirus to suppress or overexpress circ_0006156 was purchased from GeneCham (Shanghai, China). PC3 and DU145 cells were infected with the indicated viral particles according to the manufacturer’s instructions. The sequences of siRNAs can be seen in Supplemental Table 2.

### Actinomycin D and RNase R treatment assay

Cells in logarithmic growth phase were seeded in a six-well plate. Cells were treated with 5 μg/mL actinomycin D and collected at the indicated time points after 24 h. Total RNA (2 μg) with 3 U/μg RNase R (Lucigen, USA) was incubated at 37 °C for 15 min. Finally, qRT–PCR was performed to analyze the RNA expression level of circ_0006156.

### Cell counting kit-8 (CCK8) and colony formation assay

DU145 or PC3 cells (3 × 10^3^) were seeded into 96-well plates and incubated for 24, 48, 72, and 96 h. Then, 10 μL of Cell Counting Kit-8 (CCK-8; Yeasen) reagent was added to each well and incubated for another 1 h. Subsequently, the absorbance values were measured at 450 nm.

For the colony formation assay, 1000 cells/well of the indicated cells were seeded into a 6-well plate and cultured in an atmosphere of 5% CO_2_ at 37 °C for 10 days. Cells were then fixed with methanol and stained with crystal violet.

### Transwell assay

Cell invasion and migration assays were performed using 24-well Transwell cell culture chambers (Corning, USA) with or without Matrigel (Corning). Cells (8 × 10^4^) suspended in 200 μL of medium without FBS were added to the upper chamber, and 600 μL of medium containing 10% FBS was added to the bottom chamber. After incubation at 37 °C and 5% CO_2_ for 24 h (48 h for invasion assay), the cells on the lower surface of the chamber were stained with crystal violet for 30 min. The migrated and invaded cells per chamber were counted in three randomly selected fields, and independent experiments were performed in triplicate. Images were acquired using a brightfield microscope (100x magnification), and cells were counted by ImageJ (version 1.47).

### Wound-healing assay

Cells were seeded into 6-well plates, and equidistant scratches were made with a pipette tip. After culturing cells in medium without FBS for 24 h, images were acquired.

### Fluorescence in situ hybridization (FISH) assay

An oligonucleotide-modified probe of circ_0006156 was designed by GenePharma (Shanghai, China). Fixed cells were washed in PBS and treated with RNase R at 37 °C for 15 min. The cell suspension was transferred to a glass slide and then dehydrated with a gradient of 70, 80, and 100% ethanol. Hybridization was then performed overnight at 37 °C in a dark and humid chamber. After being washed twice with 50% formamide/2 × SSC for 5 min, the slides were incubated with Alexa FluorTM 488 Tyramide SuperBoost™ Kits (Thermo Fisher Scientific, USA) for 30 min and then sealed with parafilm containing DAPI. A fluorescence microscope was used to acquire images. The sequence of FISH probe can be seen in Supplemental Table 2.

### RNA pull-down assay and mass spectrometry analysis

A biotin probe targeting the back-splicing site of circ_0006156 was designed by GenePharma (China). The probe was incubated with extracts from the indicated cells at room temperature for 2 h, and Streptavidin C1 magnetic beads (35 μL, Invitrogen) were then added for 1 h. After washing, the protein was detected by Western blot and mass spectrometry analyses (Bioprofile, China). The sequences of probes can be seen in Supplemental Table 2.

### RNA-binding protein immunoprecipitation (RIP) assay

The RIP assay was performed according to the protocol of the Magna RIP Kit (Millipore, USA). In brief, DU145 cells were harvested after 48 h and lysed in RIP lysis buffer on ice for 30 min. After centrifugation, the supernatant was incubated with 30 μL of magnetic beads and the indicated antibodies. After overnight incubation, the immune complex was centrifuged and then washed six times with washing buffer. The immunoprecipitated RNA was analyzed by qRT–PCR.

### Silver-staining assay

The silver-staining assay was performed according to the protocol of the Protein Silver Stain Kit (Yeasen).

### Immunohistochemistry (IHC) staining

Tissues were fixed with 4% paraformaldehyde, dehydrated, embedded in paraffin and sectioned at 4 μm. The sections were deparaffinized, rehydrated and incubated with 3% H_2_O_2_. After antigen retrieval and blocking, the slides were incubated with the indicated antibodies (1:200) (Abcam, USA) at 4 °C overnight. Subsequently, the slides were incubated with the corresponding secondary antibody for 30 min at room temperature and then incubated with the streptavidin peroxidase complex. Staining was performed using a 3,3-diaminobenzidine (DAB) substrate kit for peroxidase reaction and counterstained with hematoxylin. Finally, a light microscope was used to acquire images.

### In vivo experiments

This study was approved by Institutional Animal Care and Use Committee of Nanjing Medical University (IACUC-2012007). Twenty-four healthy 6-week-old BALB-C nude mice purchased from Nanjing Medical University (Nanjing, China) were randomly divided into 4 groups of 6 mice each. Approximately 1 × 10^7^ of the indicated cells washed with PBS were injected into the tail vein of each mouse at one time point. Tumor nodules and progression were monitored every 2 weeks after injection. After 8 weeks of injection, all mice were sacrificed under general anesthesia (induced using an intraperitoneal injection of pentobarbital sodium (150 mg/kg)), and the tumors were removed for subsequent analyses.

### Statistical analysis

Data are presented as the mean ± SD. Paired or nonpaired two-tailed *t*-tests were used for comparisons between two groups. R software (version 4.1.0) was used for statistical analysis. *p* < 0.05 was considered significant.

## Results

### Circ_0006156 is expressed at low levels in PCa tissues and cells

**Fig. 1:**
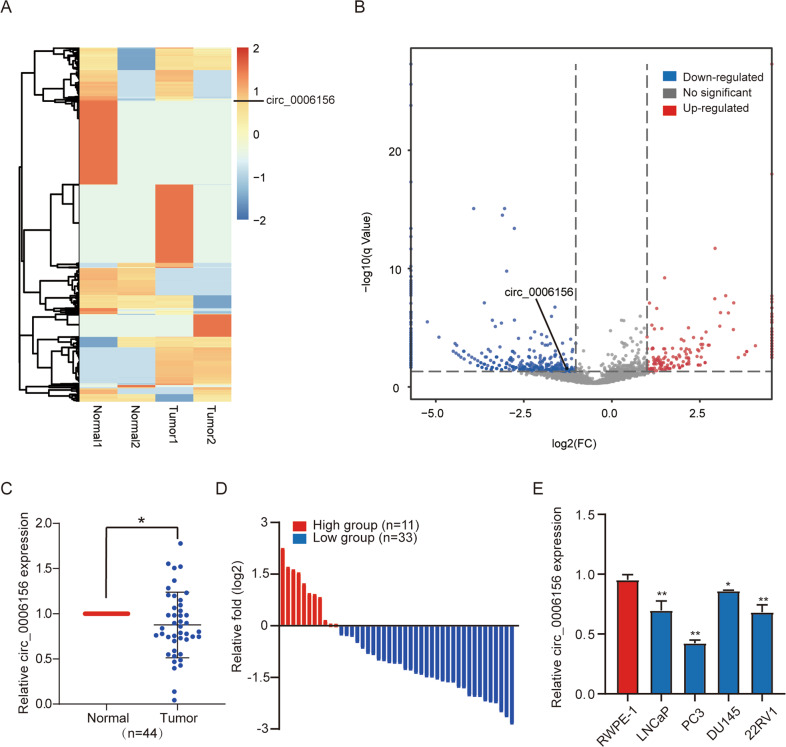
Circ_0006156 is expressed at low levels in PCa tissues and cells. **A**, **B** Heatmap and volcano map of circ_0006156 in two pairs of PCa tissues and corresponding normal tissues. **C** qRT–PCR detected the circ_0006156 expression profile in 44 pairs of PCa tissues and corresponding normal tissues. **D** The expression of circ_0006156 was significantly low in 75% of PCa patients. **E** qRT–PCR detected the circ_0006156 expression profile in PCa cell lines compared to normal RWPE-1 cells.

To investigate circRNA alterations in PCa, we performed high-throughput circRNA sequencing using two pairs of PCa and matched adjacent nontumor tissue samples. A total of 2111 differentially expressed circRNAs were identified (details were shown in Supplemental Table 1), among which circ_0006156 was found to be expressed at low levels in PCa tissues (Fig. 1A, B). Next, we compared the expression of circ_0006156 in PCa tissues to that in matched normal tissues from 44 PCa patients, and we found that the expression of circ_0006156 was low in 75% of PCa patients (Fig. 1C, D). To characterize the expression profile of circ_0006156 in PCa cell lines, qRT–PCR analyses were conducted in the normal human prostate epithelial cell line, RWPE-1, and four PCa cell lines, namely, LNCaP, PC3, DU145 and 22RV1. The results showed that compared to RWPE-1 cells, the abundance of circ_0006156 was markedly decreased in all four PCa cell lines (Fig. 1E). We selected DU145 cells with the highest relative expression and PC3 with the lowest relative expression for subsequent experiments. We knocked down circ_0006156 in DU145 cells, and we overexpressed circ_0006156 in PC3 cells.

### Characterization of circ_0006156 in PCa cells

**Fig. 2:**
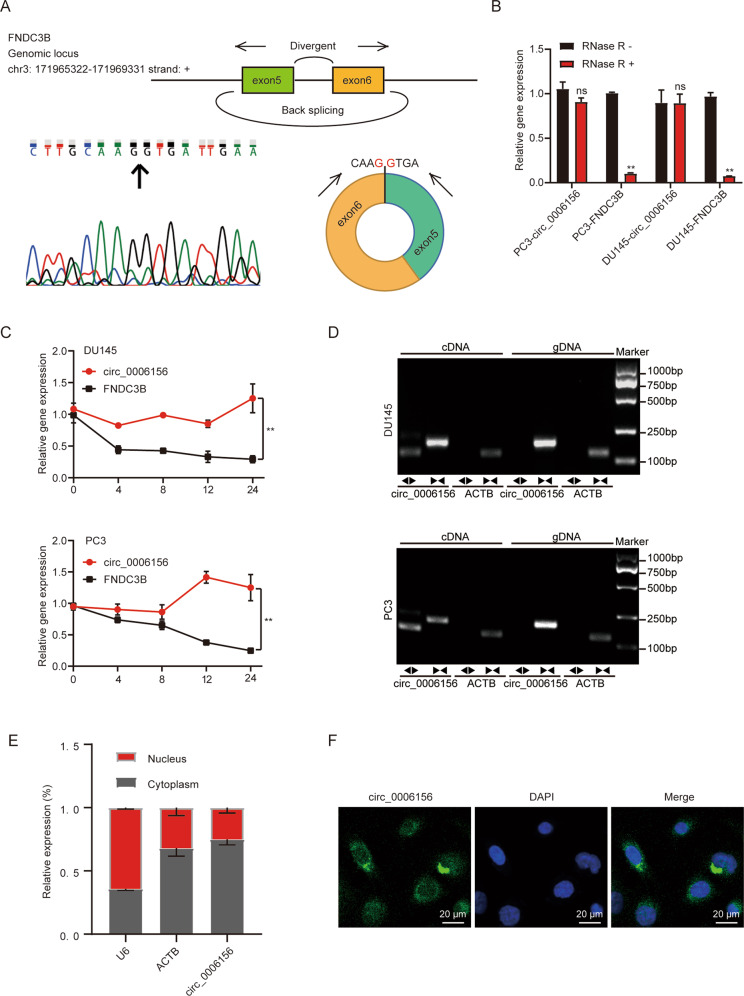
Characteristics of circ_0006156 in PCa cells. **A** Genomic locus of circ_0006156. The expression of circ_0006156 was detected by qRT–PCR followed by Sanger sequencing. Arrows represent divergent primers binding to the back-splicing site of circ_0006156. **B** qRT–PCR analysis of the expression of circ_0006156 and FNDC3B mRNA after treatment with RNase R in PCa cells. **C** qRT–PCR analysis of the expression of circ_0006156 and FNDC3B mRNA after treatment with actinomycin D at the indicated time points in PCa cells. **D** qRT–PCR products with divergent primers showing circularization of circ_0006156 in PCa cells. cDNA, complementary DNA. gDNA, genomic DNA. **E** Cytoplasmic and nuclear mRNA fractionation experiments showed that circ_0006156 localized in both the nucleus and cytoplasm in DU145 cells. ACTB and U6 were applied as positive controls in the cytoplasm and nucleus, respectively. **F** RNA fluorescence in situ hybridization for circ_0006156 in DU145 cells. Nuclei were stained with DAPI.

Circ_0006156 is back-spliced from 2 exons (exons 5 and 6) of the *FNDC3B* gene. The loop structure was confirmed by Sanger sequencing (Fig. 2A), and the circular structure of circ_0006156 was further confirmed in PCa cells. Circ_0006156 was resistant to RNase R or ActD treatment (Fig. 2B, C), and it was only amplified by divergent primers in cDNA (Fig. 2D). In addition, circ_0006156 was present in both the cytoplasm and nucleus of DU145 cells (Fig. 2E, F). These results collectively revealed that circ_0006156 is a stable circRNA expressed in PCa.

### Circ_0006156 suppresses the migration and invasion of PCa cells

**Fig. 3:**
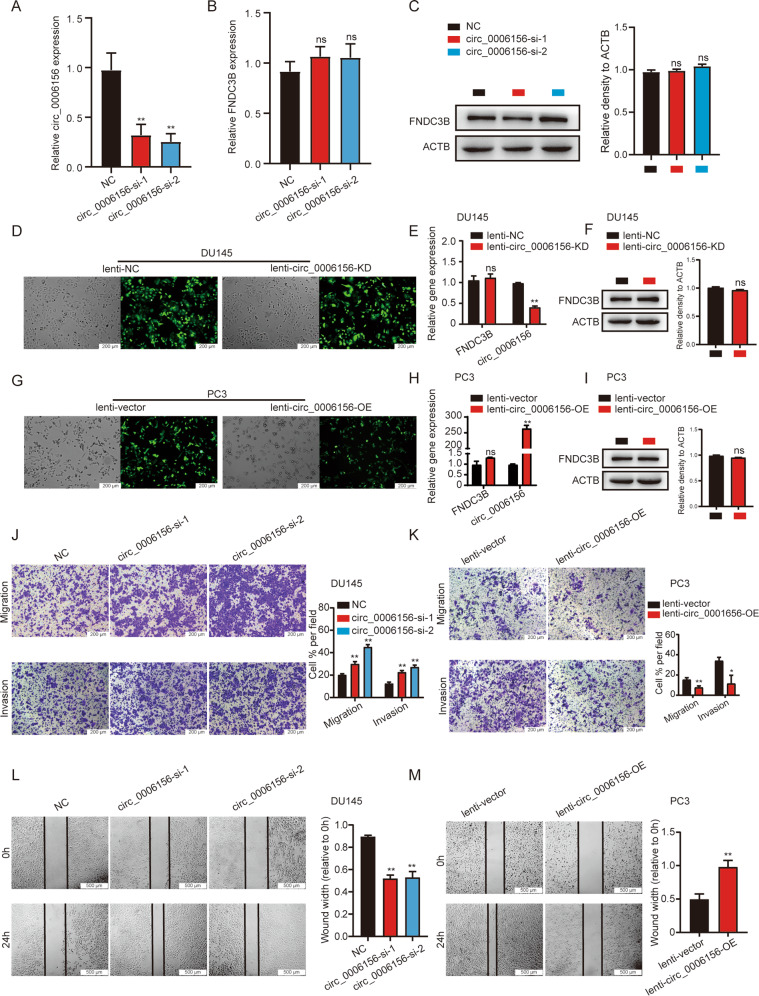
Circ_0006156 suppresses the migration and invasion of PCa cells. **A**–**C** qRT–PCR and western blot analyses of the specific inhibition efficiency of circ_0006156 in PCa cells. **D** Fluorescence showed the efficiency of lentivirus infection in DU145 cells. **E**, **F** qRT–PCR and western blot analyses of the specific inhibition efficiency of circ_0006156 in DU145 cells. **G** Fluorescence showed the efficiency of lentivirus infection in PC3 cells. **H**, **I** qRT–PCR and western blot analyses of the specific overexpression efficiency of circ_0006156 in PC3 cells. **J**, **K** Transwell assays showed that circ_0006156 suppressed the migration and invasion of PCa cells. **L**, **M** Wound-healing assays showed that circ_0006156 weakened the migration of PCa cells.

To investigate the impact of circ_0006156 on PCa cell functions, we knocked down and overexpressed circ_0006156 in PCa cells. Both siRNAs targeting the back-splicing site of circ_0006156 achieved over 50% knockdown levels in PCa cells with no significant effect on FNDC3B mRNA or protein (Fig. 3A–C). We selected siRNA-2 to generate a lentivirus for subsequent experiments (Fig. 3D–F), and we constructed an overexpression lentivirus to stably overexpress circ_0006156 (Fig. 3G–I). When circ_0006156 was knocked down, the migration and invasion abilities of DU145 cells significantly increased (Fig. 3J). Moreover, PC3 cell migration and invasion were suppressed when circ_0006156 was overexpressed (Fig. 3K). The same results were observed in wound-healing assays in PCa cells (Fig. 3L, M). In addition, we analyzed the influence of circ_0006156 on the proliferation ability of PCa cells via CCK-8 and colony formation assays. However, the results revealed no significant effect of circ_0006156 on PCa cell proliferation (Supplemental Fig. 1A–D). Taken together, these data demonstrated that circ_0006156 is negatively correlated with aggressive phenotypes of PCa cells.

### Circ_0006156 binds to and stabilizes S100A9 by blocking ubiquitination

**Fig. 4:**
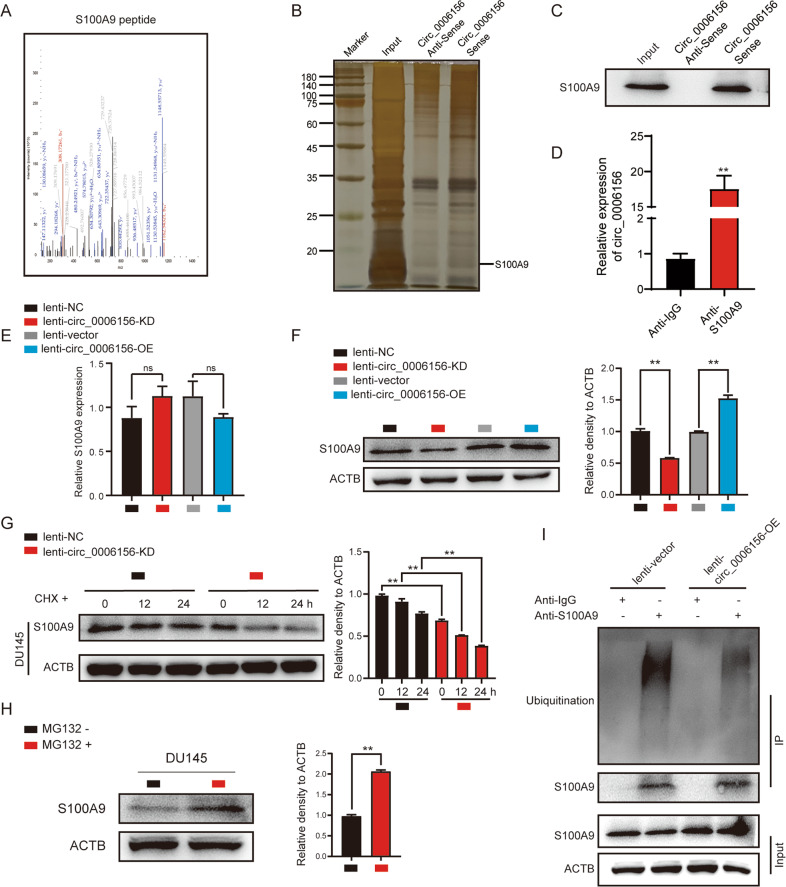
Circ_0006156 binds to and stabilizes S100A9 by blocking ubiquitination. **A** The secondary mass spectrum result of S100A9 peptide. **B**, **C** Silver-staining assay after RNA pulldown showed that S100A9 binds to circ_0006156. **D** RIP assay showed that circ_0006156 binds to S100A9 in DU145 cells. **E**, **F** qRT–PCR and Western blot analyses showed that S100A9 protein, but not mRNA, was influenced after circ_0006156 knockdown or overexpression. **G** Western blot analysis of S100A9 expression after treatment with cycloheximide at the indicated time points in different groups. **H** S100A9 expression was increased after treatment with MG132. **I** Western blot analysis after co-IP showed that the ubiquitination level of S100A9 was decreased after overexpressing circ_0006156.

We next investigated the molecular mechanism by which circ_0006156 weakens PCa migration and invasion. Mass spectrometry was performed following an RNA pull-down assay, and we found that 80 potential proteins may specifically bind to circ_0006156 (Supplemental Table 3). The Human Cancer Metastasis Database [20] (HCMDB, https://hcmdb.i-sanger.com/index) is an integrated database designed to store and analyze large-scale expression data of cancer metastasis. Considering that circ_0006156 significantly inhibits PCa cells migration and invasion, we analyzed these 80 proteins in the HCMDB for proteins important in PCa metastasis and we finally selected S100A9 as a potential target for further verification. Detailed information on S100A9 from the mass spectrometric analysis is shown in Fig. 4A. RNA pulldown, Western blot and silver staining assays indicated the binding relationship between circ_0006156 and S100A9 (Fig. 4B, C). RIP assays further revealed that S100A9 protein specifically binds to circ_0006156 (Fig. 4D).

We then investigated the influence of circ_0006156 on S100A9. qRT–PCR analysis showed that the mRNA level of S100A9 was not significantly affected in response to circ_0006156 interference or enhancement in PCa cells (Fig. 4E). However, the protein expression of S100A9 was positively regulated by circ_0006156 overexpression and negatively regulated by circ_0006156 knockdown (Fig. 4F), suggesting that circ_0006156 improves S100A9 protein stability. Cycloheximide (CHX) was then used to analyze the stability of proteins. Western blot analysis showed that the expression level of S100A9 decreased with circ_0006156 interference at the indicated time points in DU145 cells (Fig. 4G), which revealed the positive influence of circ_0006156 on S100A9 protein stability.

Ubiquitination is an important regulatory mechanism that affects protein stability [21]. Therefore, we treated PCa cells with MG132 (ubiquitination inhibitor) for 6 h, and Western blot analysis demonstrated that the protein expression level of S100A9 was significantly increased (Fig. 4H). Furthermore, we searched the HitPredict Database [22–24] (http://www.hitpredict.org) for proteins that may bind to S100A9 and found 8 E-3 ubiquitin-protein ligases with high confidence (Supplemental Fig. 2), suggesting that circ_0006156 may regulate S100A9 protein through a ubiquitination degradation pathway. Therefore, a co-IP assay was performed in PC3 cells (Fig. 4I). The results showed that overexpression of circ_0006156 significantly reduced the ubiquitination level of S100A9 protein. Taken together, these findings demonstrated that circ_0006156 binds to and stabilizes S100A9 by blocking ubiquitination.

### S100A9 suppresses the migration and invasion of PCa cells, and overexpression of S100A9 rescues this effect after knockdown of circ_0006156

We first detected the expression levels of S100A9 in PCa cells and tissues. qRT–PCR assays identified that S100A9 was at low expression levels in PCa cells and tissues (Supplementary Fig. 3A–C). We next analyzed data from the TCGA database (https://portal.gdc.cancer.gov/) and found that S100A9 was downregulated in PCa tissues compared to normal tissues (Supplemental Fig. 3D). The K-M plot of S100A9 from the ATLAS database [25–27] (https://www.proteinatlas.org/) revealed a positive relationship between S100A9 expression and the survival time of PCa patients (Supplemental Fig. 3E). We further searched the HCMDB database for data related to S100A9 and PCa metastasis, and we found that the expression of S100A9 was significantly decreased in bone metastatic PCa (Supplemental Fig. 3F, G).

**Fig. 5:**
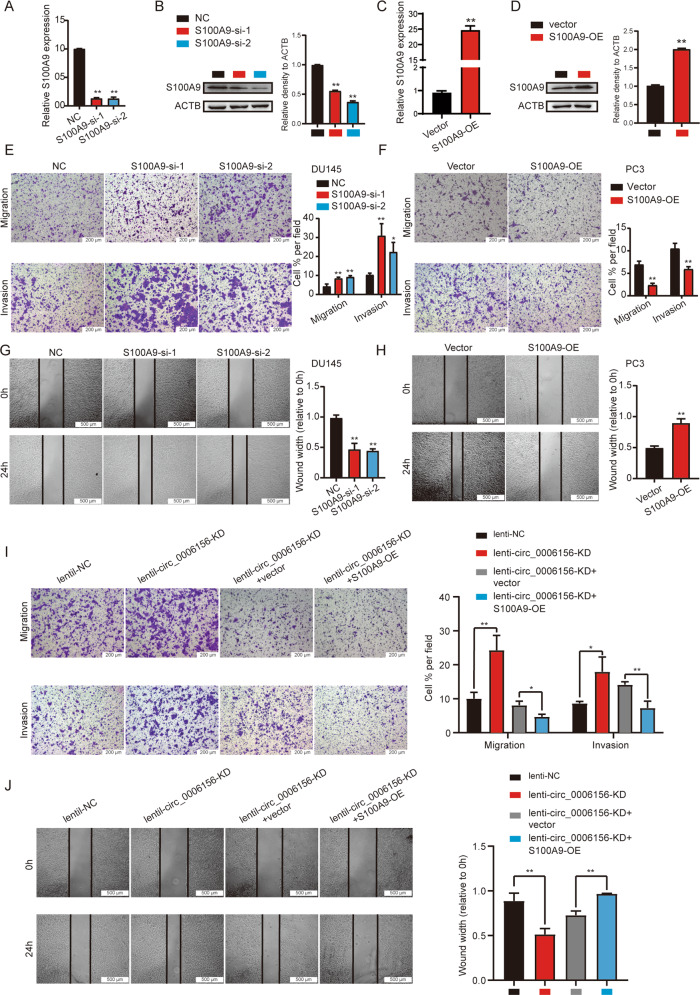
S100A9 suppresses the migration and invasion of PCa cells, and overexpression of S100A9 rescues this effect after knockdown of circ_0006156. **A**–**D** qRT–PCR and western blot analyses of the inhibition and overexpression efficiency of S100A9 in PCa cells. **E**, **F** Transwell assays showed that S100A9 suppressed the migration and invasion of PCa cells. **G**, **H** Wound-healing assays showed that S100A9 weakened the migration of PCa cells. **I**, **J** Transwell and wound-healing assays showed that the migration and invasion abilities enhanced by knocking down circ_0006156 were partially inhibited by overexpressing S100A9.

To investigate the effect of S100A9 on PCa migration and invasion, we knocked down and overexpressed S100A9 in PCa cells (Fig. 5A–D). Silencing S100A9 markedly promoted PCa cell migration and invasion (Fig. 5E, G), and S100A9 overexpression reversed this effect (Fig. 5F, H).

Rescue assays were then performed, which demonstrated that overexpression of S100A9 significantly weakened the enhancement of PCa cell migration and invasion caused by knocking down circ_0006156 (Fig. 5I, J). Taken together, these findings demonstrated that circ_0006156 stabilizes S100A9 protein by blocking ubiquitination and inhibits PCa cell migration and invasion by binding to S100A9.

### Circ_0006156 inhibits PCa metastasis in vivo

**Fig. 6:**
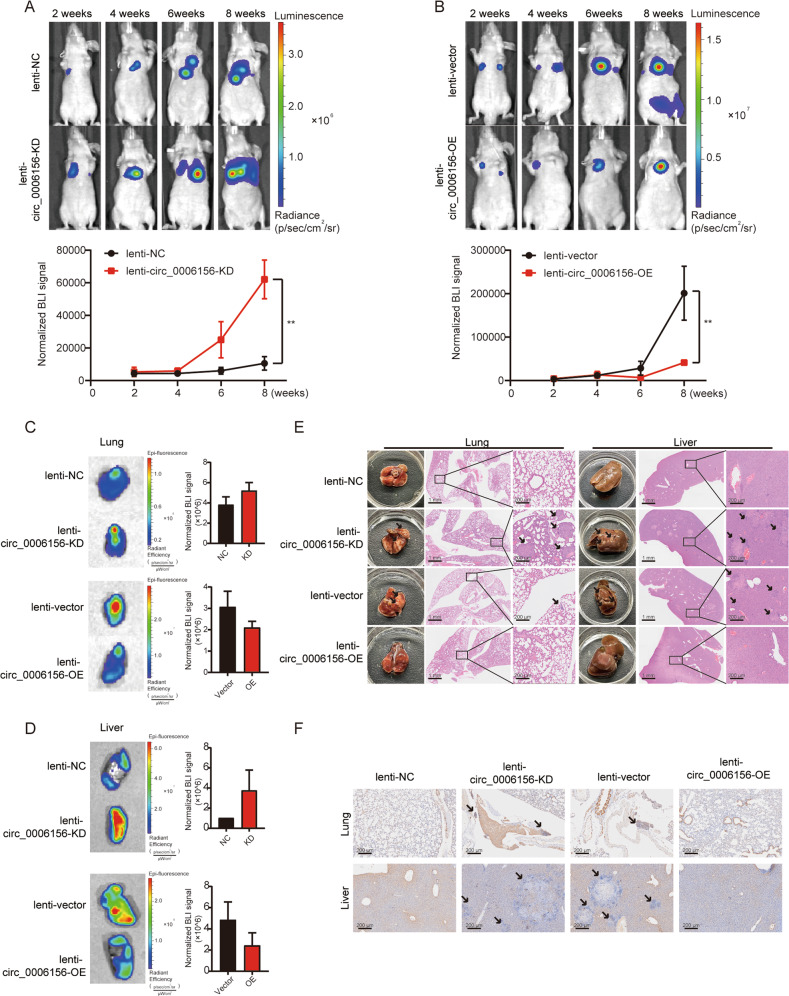
Circ_0006156 inhibits PCa metastasis in vivo. **A**, **B** Upper panels: representative bioluminescent (BLI) images acquired at the indicated time points after injection with different groups of PCa cells. Pseudocolor heatmaps indicate the intensity of bioluminescence from low (blue) to high (red). Lower panels: normalized BLI signals of tumors of corresponding mice recorded at the indicated time points. **C**, **D** Representative BLI images and normalized BLI signals of lungs and livers acquired from the indicated mice. **E** Representative images of lung and liver nodules in the indicated groups. **F** Immunohistochemistry was used to analyze S100A9 expression in the indicated groups.

In vivo experiments were performed to further validate the role of circ_0006156 in PCa. Luminescence increased significantly after knocking down circ_0006156 at the indicated time points, and the opposite result was observed after overexpression of circ_0006156 (Fig. 6A, B). The epifluorescence of liver and lung tissues was strong in the circ_0006156-deficient groups but weak in the circ_0006156 overexpression groups (Fig. 6C, D). The colonization of PCa cells was significantly enhanced as indicated by the increased number of tumor nodules in mice that received circ_0006156-deficient cells, and the opposite results were found with circ_0006156-overexpressing cells (Fig. 6E). Additionally, the expression level of S100A9 was lower in circ_0094606-deficient xenografts but higher in circ_0094606-overexpressing xenografts (Fig. 6F). Altogether, these findings indicated that circ_0006156 inhibits PCa cell metastasis by stabilizing S100A9 in vivo.

## Discussion

PCa is one of the most prevalent malignant cancers worldwide and severely endangers human health [28]. Several studies have identified that the development of PCa involves a variety of gene expression disorders [29] with circRNAs playing an important role in regulating the expression of genes related to the occurrence and progression of PCa [30, 31]. In depth research on the biological functions of circRNAs in tumors has suggested that there are three main mechanisms as follows: (1) ceRNA regulator network, in which circRNAs sponge miRNAs to regulate target genes; [32] (2) few circRNAs have been identified to translate peptides, which is different from the characterization of noncoding RNAs; [33, 34], and (3) some circRNAs regulate gene expression at the posttranscriptional level [35].

Circ_0006156, a tumor-specific circRNA, has been reported to play different roles in a variety of tumors. For instance, He et al. [36] reported that the expression of circ_0006156 is downregulated in gastric cancer tissues and is related to the differentiation degree, presence or absence of lymph node metastasis and prognosis of gastric cancer patients. Zeng et al. [37] showed that circ_0006156 sequesters miR-937-5p to derepress TIMP3 and inhibits colorectal cancer progression. However, some studies have revealed the positive effect of circ_0006156 on the occurrence and progression of several cancers. Wu et al [38]. demonstrated that circ_0006156 promotes papillary thyroid cancer progression through the miR-1178/TLR4 pathway. Hong et al. [39] identified that circ_0006156 enhances the migration and invasion of gastric cancer cells via the regulation of E-cadherin and CD44 expression. Although many studies have reported the important functions of circ_0006156 in tumors, it remains unknown whether it plays a biological function in PCa. In the present study, we found that circ_0006156 blocks the ubiquitination of S100A9 to stabilize S100A9 and inhibit the metastasis of PCa.

The S100 family is a group of proteins that is emerging as a potentially vital group of markers in multiple tumor types, and altered expression of the S100 family has been observed in several cancers, including breast, lung, gastric and prostate cancers [40]. S100 proteins are commonly upregulated in tumors and are often positively associated with tumor progression. However, some members of the S100 family have been reported to be tumor suppressors, including S100A2, S100A11, and S100A9 [40–42]. For instance, Gumireddy et al. [43] identified that Id1 promotes breast cancer metastasis by suppressing S100A9 expression. Choi et al. [44] reported that increased expression of stromal S100A9 in gastric adenocarcinoma is associated with small lesion size and a decrease in lymph node metastasis. Yun et al. [45] reported lower expression of S100A9 in PCa tissues than in benign prostatic hyperplasia tissues.

In the present study, we found for the first time that circ_0006156 binds to and stabilizes S100A9, thereby inhibiting the metastasis of PCa. Collectively, these findings demonstrated that circ_0006156 plays a vital role in PCa, suggesting that circ_0006156 may be a potential therapeutic target and companion biomarker for PCa.

## Supplementary information

Supplemental Figures

Supplemental legends

Supplemental Table 1

Supplemental Table 2

Supplemental Table 3

## Data Availability

The data of this study are available from the corresponding author on reasonable request.
