# Supplementary material for: CircRNA circ_0006156 inhibits the metastasis of prostate cancer by blocking the ubiquitination of S100A9
Source: Cancer Gene Ther. 2022 Jun 27;29(11):1731–41. doi: 10.1038/s41417-022-00492-z (PMC9663304; doi:10.1038/s41417-022-00492-z)
Supplement: Supplementary file 1 — Supplemental Figures [file 41417_2022_492_MOESM1_ESM.docx]

**CircRNA circ_0006156 inhibits the metastasis of prostate cancer by blocking the ubiquitination of S100A9**


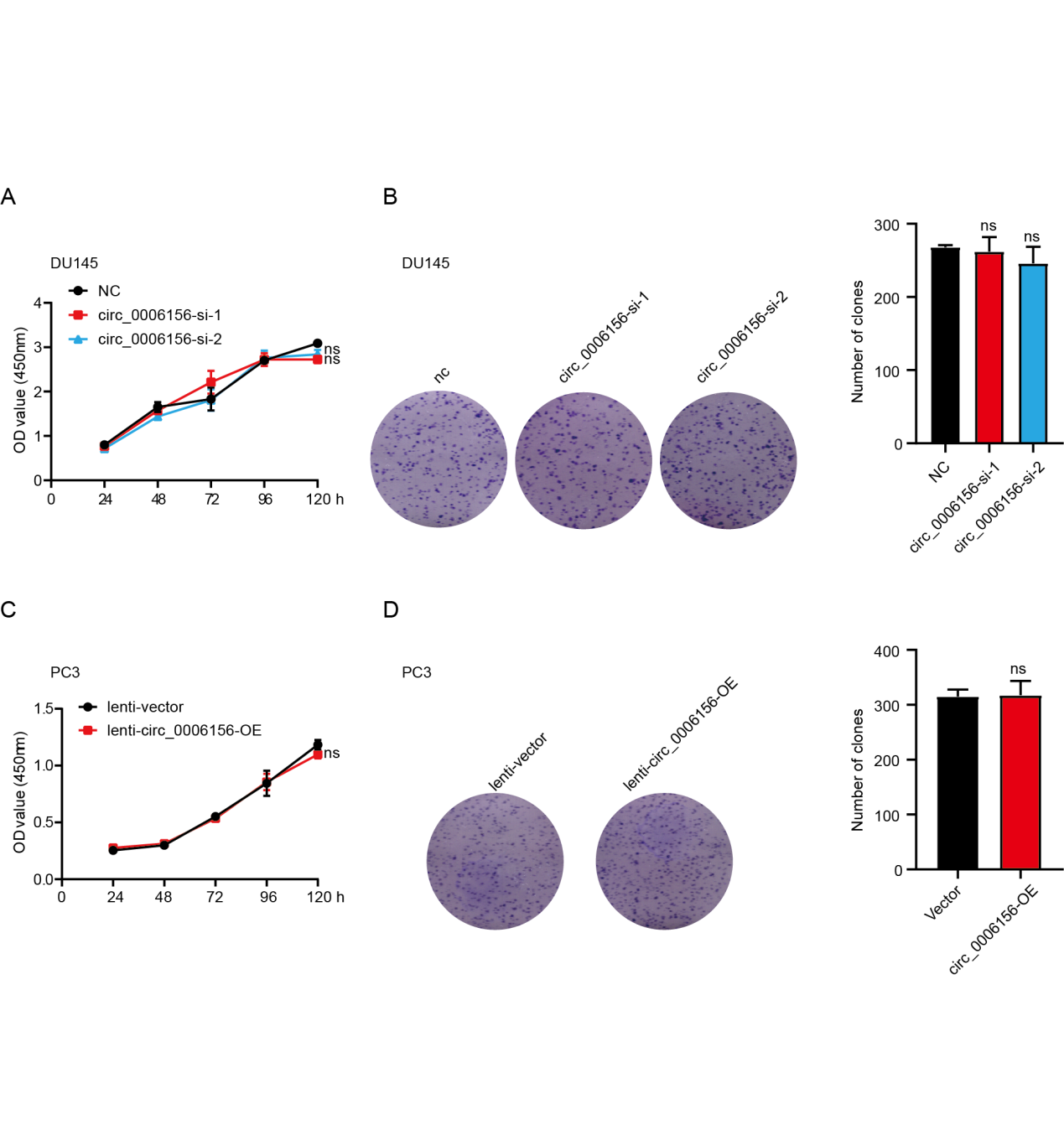


**Supplemental Figure 1. Circ_0006156 does not affect the proliferation ability of PCa cells.** (A-B) CCK-8 and colony formation assays showed that the proliferation ability of DU145 cells was not significantly changed after knocking down circ_0006156. (C-D) CCK-8 and colony formation assays showed that the proliferation ability of PC3 cells was not significantly changed after overexpressing circ_0006156. ns means no significance.


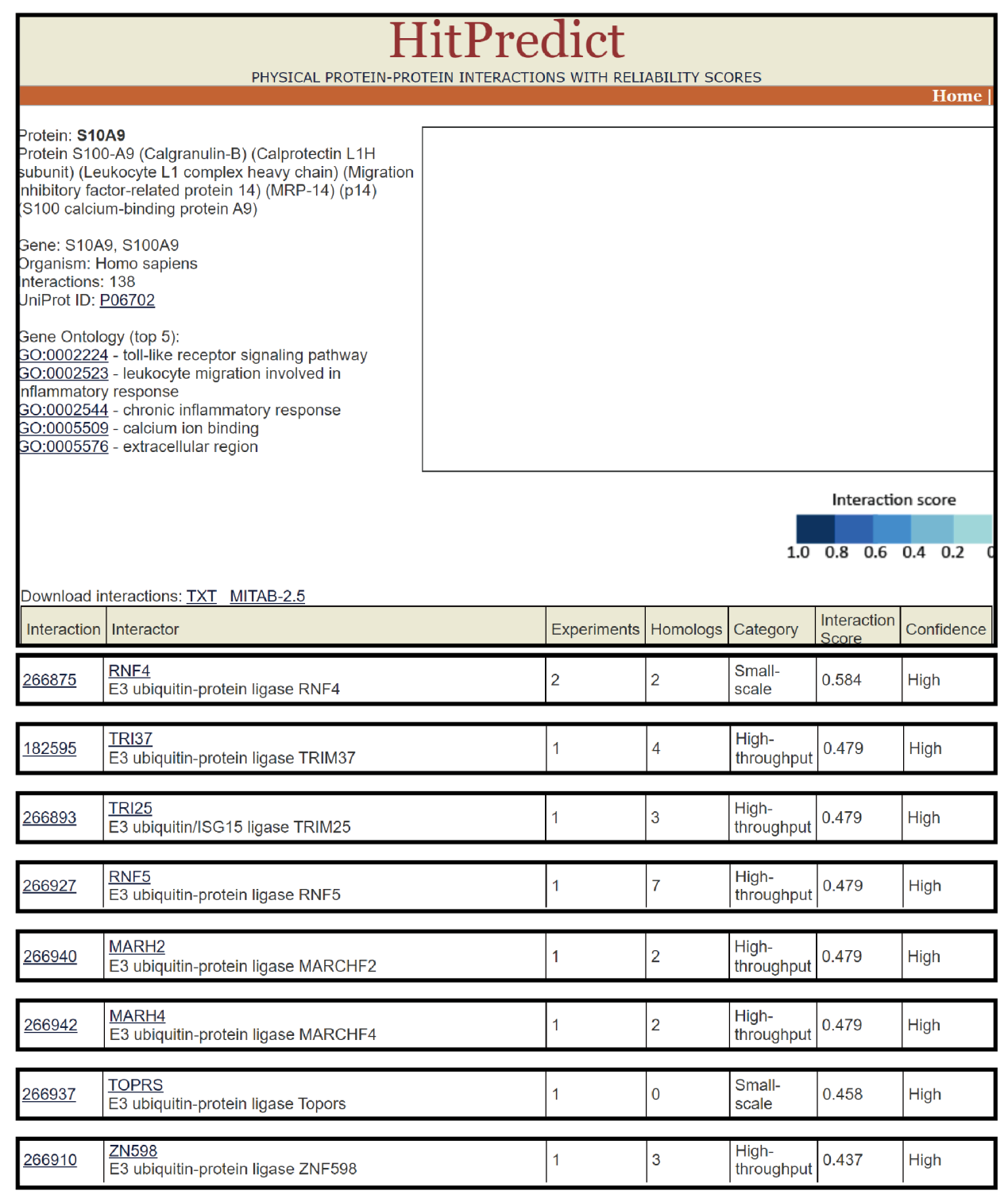


**Supplemental Figure 2. Potential E-3 ubiquitin-protein ligases of S100A9 predicted by the HitPredict Database.**

**
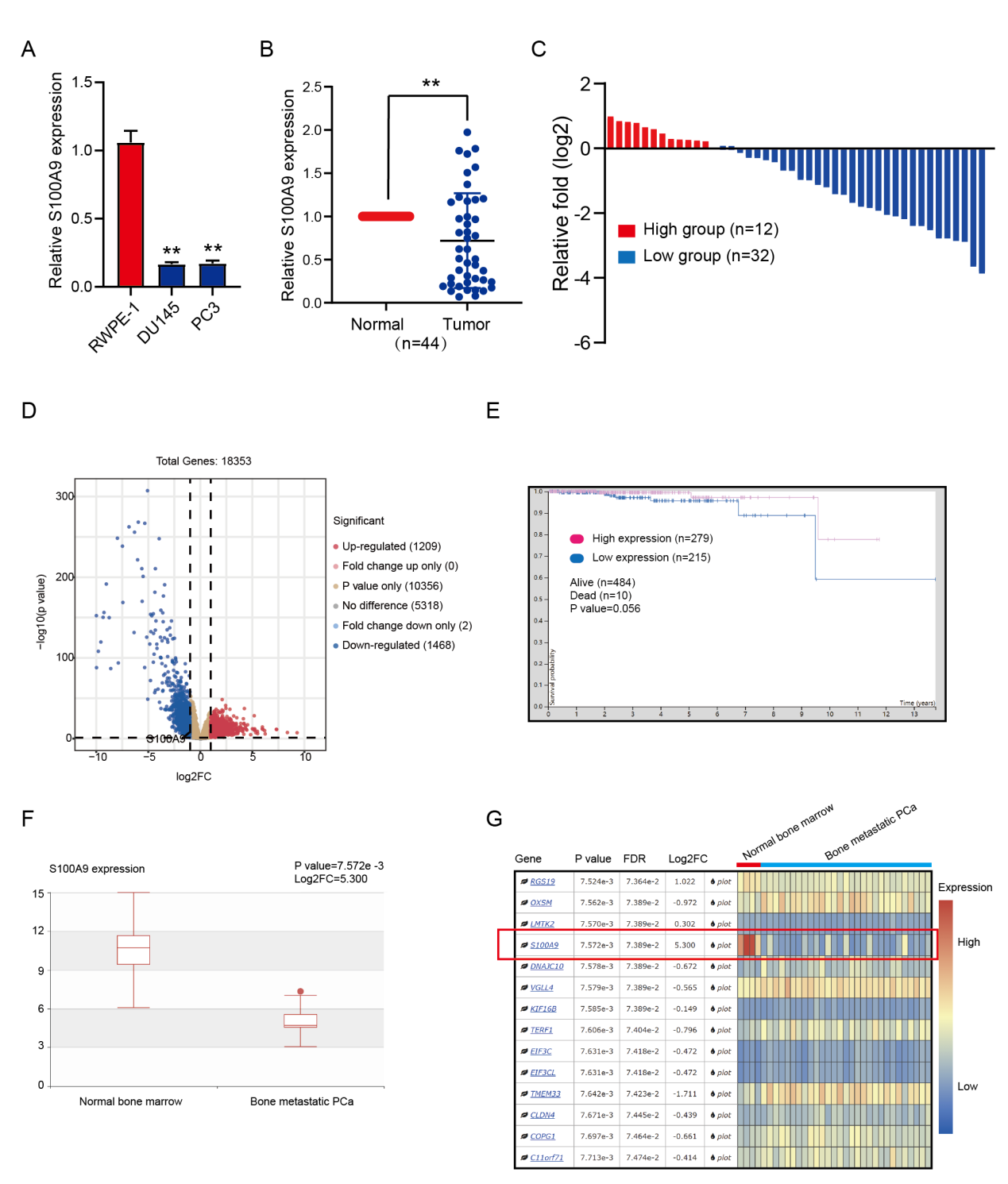
**

**Supplemental Figure 3. S100A9 is expressed at low levels in PCa cells and tissues.** (A) S100A9 was expressed at low levels in PCa cells. (B) qRT-PCR detected the S100A9 expression profile in 44 pairs of PCa tissues and corresponding normal tissues. (C) The expression of S100A9 was significantly low in nearly 73% of PCa patients. (D) S100A9 was down-regulated in PCa tissues compared to normal tissues from the TCGA database. (E) K-M survival plot of S100A9 from the ATLAS database. (F-G) Expression of S100A9 in the indicated groups from the HCMDB database. ^**^*P*<0.01.
