# Supplementary material for: CircRNA circ_0006156 inhibits the metastasis of prostate cancer by blocking the ubiquitination of S100A9
Source: Cancer Gene Ther. 2022 Jun 27;29(11):1731–41. doi: 10.1038/s41417-022-00492-z (PMC9663304; doi:10.1038/s41417-022-00492-z)
Supplement: Supplementary file 2 — Supplemental legends [file 41417_2022_492_MOESM2_ESM.docx]

**CircRNA circ_0006156 inhibits the metastasis of prostate cancer by blocking the ubiquitination of S100A9**

**Supplemental legends**

**Supplemental Table 1. The circRNA sequencing data of two pairs of PCa and matched adjacent non-tumor tissue samples.**

**Supplemental Table 2. The primers used in the present study.**

**Supplemental Table 3. Data of the mass spectrometry.**
